# Engineered Antibodies as Cancer Radiotheranostics

**DOI:** 10.1002/advs.202402361

**Published:** 2024-06-14

**Authors:** Zhenni Wei, Bingyu Li, Xuejun Wen, Vivianne Jakobsson, Peifei Liu, Xiaoyuan Chen, Jingjing Zhang

**Affiliations:** ^1^ Department of Diagnostic Radiology, Yong Loo Lin School of Medicine National University of Singapore Singapore 119074 Singapore; ^2^ Nanomedicine Translational Research Program NUS Center for Nanomedicine Yong Loo Lin School of Medicine National University of Singapore Singapore 117597 Singapore; ^3^ Clinical Imaging Research Centre Centre for Translational Medicine Yong Loo Lin School of Medicine National University of Singapore Singapore 117599 Singapore; ^4^ Theranostics Center of Excellence (TCE) Yong Loo Lin School of Medicine National University of Singapore 11 Biopolis Way, Helios Singapore 138667 Singapore; ^5^ Departments of Surgery Chemical and Biomolecular Engineering and Biomedical Engineering Yong Loo Lin School of Medicine and College of Design and Engineering National University of Singapore Singapore 119074 Singapore; ^6^ Institute of Molecular and Cell Biology Agency for Science Technology and Research (A*STAR) 61 Biopolis Drive, Proteos Singapore 138673 Singapore

**Keywords:** antibodies, cancer radiotheranostics, engineered antibodies, immunoPET, radioimmunotherapy

## Abstract

Radiotheranostics is a rapidly growing approach in personalized medicine, merging diagnostic imaging and targeted radiotherapy to allow for the precise detection and treatment of diseases, notably cancer. Radiolabeled antibodies have become indispensable tools in the field of cancer theranostics due to their high specificity and affinity for cancer‐associated antigens, which allows for accurate targeting with minimal impact on surrounding healthy tissues, enhancing therapeutic efficacy while reducing side effects, immune‐modulating ability, and versatility and flexibility in engineering and conjugation. However, there are inherent limitations in using antibodies as a platform for radiopharmaceuticals due to their natural activities within the immune system, large size preventing effective tumor penetration, and relatively long half‐life with concerns for prolonged radioactivity exposure. Antibody engineering can solve these challenges while preserving the many advantages of the immunoglobulin framework. In this review, the goal is to give a general overview of antibody engineering and design for tumor radiotheranostics. Particularly, the four ways that antibody engineering is applied to enhance radioimmunoconjugates: pharmacokinetics optimization, site‐specific bioconjugation, modulation of Fc interactions, and bispecific construct creation are discussed. The radionuclide choices for designed antibody radionuclide conjugates and conjugation techniques and future directions for antibody radionuclide conjugate innovation and advancement are also discussed.

## Graphical abstract

This review examines radiotheranostics in personalized medicine, combining diagnostic imaging and targeted radioimmunotherapy for effective cancer treatment. It highlights the use of radiolabeled antibodies for precise delivery, the challenges in radiopharmaceutical applications, and the promise of antibody engineering to enhance treatment efficacy and safety. It also considers radionuclide selection, conjugation methods, and future innovations in antibody radionuclide conjugates.

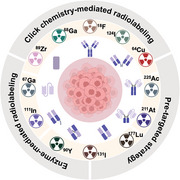

## 1 Introduction

The goal of the quickly emerging medical area of theranostics is to integrate therapy and imaging into a single platform for use in the next wave of customized medicine.^[^ ^1^ ^]^ This strategy relies on imaging to validate the existence of a biological target, followed by therapeutic actions. Despite the decades‐long combination of imaging and therapy, the field has made significant strides in recent years.^[^ ^2^ ^]^ The cornerstone of theranostics, radiotheranostics, radiolabeled compounds that can both visualize and eradicate targeted cells, facilitating a dual role in patient management by enabling the assessment of disease presence and the subsequent targeted therapeutic intervention, has significantly enhanced healthcare worldwide since its introduction into clinical practice. Selecting patients for targeted radiotherapy based on imaging of the same target region is a crucial aspect of radiotheranostics, which relies on the premise that the uptake of the radiolabeled compound used for imaging mirrors the uptake of the therapeutic agent, ensuring that the treatment is delivered specifically to the areas identified by imaging.^[^ ^3^, ^4^ ^]^

One of the most important components of radiotheranostic agents is the targeting moiety, which determines the specificity, affinity, and stability of the agent for the tumor antigen. For example, radiolabeled somatostatin (SST) analogs, such as DOTATATE, have been widely used for targeting SST receptor‐positive neuroendocrine tumors;^[^ ^5^ ^]^ prostate‐specific membrane antigen (PSMA)‐targeting small molecules, such as PSMA‐617,^[^ ^6^ ^]^ are being increasingly included in prostate cancer care guidelines; and fibroblast activation protein (FAP)‐targeting moieties are heavily researched for their pan‐tumoral targeting ability.^[^ ^7^, ^8^, ^9^ ^]^ Antibodies are widely used as targeting moieties for radiotheranostics due to their high specificity and diversity;^[^ ^10^ ^]^ radiolabeled antibodies are called antibody radionuclide conjugates (ARCs). Creating ARCs involves attaching a radioactive isotope to monoclonal antibodies (mAbs), antibody fragments, or engineered variants such as bispecific antibodies. A variety of radionuclides can be used to label antibodies in both preclinical and clinical settings. These include gamma emitters for single photon emission computed tomography (SPECT) imaging, positron emitters for positron emission tomography (PET) imaging, and beta or alpha emitters for radioimmunotherapy (RIT).^[^ ^11^ ^]^ RIT has shown promise in the treatment of hematological malignancies, including leukemia and lymphoma, as well as some solid tumors, such as glioblastoma, melanoma, neuroendocrine tumors, prostate, breast, and ovarian cancer.^[^ ^12^ ^]^ RIT harnesses the specificity of antibodies to deliver radiation directly to cancer cells and their microenvironment, including tumor microenvironment, immune microenvironment, and microenvironmental cells, thereby minimizing damage to healthy tissues associated with conventional radiation therapy.

Tumor heterogeneity is now an obvious consideration in clinical disease management. Consequently, the use of ARCs in clinical settings has increased with the discovery of more tumor cell surface antigens.^[^ ^13^, ^14^ ^]^ One instance is the application of RIT in the management of non‐Hodgkin's lymphoma (NHL),^[^ ^15^ ^]^ delivering β‐emitting radionuclides to tumor cells via mAbs that recognize lymphoma‐specific surface antigens.^[^ ^15^, ^16^ ^]^ For example, Ibritumomab Tiuxetan, an anti‐CD20 antibody coupled to yttrium‐90, a β‐emitter with a half‐life of 64 h and tissue penetration of 5 mm, was the first RIT agent authorized by the FDA.^[^ ^17^ ^]^ Patients with relapsed or refractory NHL, as well as those with rituximab‐refractory NHL, have demonstrated effectiveness with ibritumomab Tiuxetan.^[^ ^18^ ^]^ Another RIT agent developed is Tositumomab, an anti‐CD20 antibody conjugated to iodine‐131, a β‐emitter with a half‐life of 8 days and a tissue penetration of 2.4 mm.^[^ ^19^, ^20^ ^]^ Tositumomab has also shown efficacy in patients with relapsed or refractory NHL, especially in those with follicular lymphoma.^[^ ^21^ ^]^

mAbs have been envisioned as ideal vehicles for delivering radionuclides to tumors ever since Pressman and Korngold pioneered this concept several decades ago.^[^ ^22^, ^23^ ^]^ This field has witnessed remarkable progress over the decades and encouraging clinical outcomes with radioimmunoconjugates labeled with ^131^I, ^225^Ac, and ^177^Lu for radioimmunotherapy and with ^89^Zr for immunoPET. However, a recurring theme in the literature on immunoPET, immunoSPECT, and radioimmunotherapy is the need to overcome the inherent drawbacks of antibodies as radiopharmaceutical vectors.^[^ ^24^ ^]^ Antibodies are not merely passive carriers of radionuclides to target cells; they are complex and multifunctional molecules that evolved as part of the immune system. Thus, using antibodies for radiotheranostics entails both benefits and challenges. On the positive side, high specificity and affinity for cancer antigens, good in vivo stability, and significant tumor uptake are all provided by radiolabeled antibodies. On the negative side, they might be difficult to synthesize in a homogeneous and well‐defined manner, have lengthy biological half‐lives, and may be unintentionally absorbed into healthy tissues. Many studies have been conducted to overcome the limitations of antibodies as radiopharmaceutical vectors and enhance their potential as therapeutics, theranostics, and diagnostics.^[^ ^25^ ^]^ Three primary paths have been taken. One method is called “in vivo pretargeting”, where the radionuclide and the antibody are given apart and given time to separate before the antibody binds to the tumor spot through an extremely specific response.^[^ ^23^ ^]^ This method reduces the dosimetry problems associated with conventional radioimmunoconjugates but adds considerable scientific and logistical complexity.^[^ ^23^ ^]^ Utilizing artificial biomolecules like scaffold polypeptides, DARPins, affibody molecules, engineered ankyrin repeat proteins, and antibody mimetics—which imitate the structure and functionality of antibodies—is a further strategy.^[^ ^26^ ^]^ These molecules often have better pharmacokinetic properties than radiolabeled antibodies but lack some of the features of IgG‐based platforms, such as bivalency, high tumor accumulation, and in vivo stability. Scaffold polypeptides' minimum immune response, DARPins' strong, high‐affinity binding and economical synthesis, and affibodies' precise targeting and low toxicity constitute a revolutionary advance in therapeutic design and implementation. In this article, we will discuss the third approach: antibody engineering.

Our aim in this review is to provide a broad overview of the design and engineering of antibodies for tumor radiotheranostics. We specifically go over the four ways that antibody engineering has been used to improve radioimmunoconjugates: pharmacokinetics optimization, bispecific construct generation, site‐specific bioconjugation, and regulation of Fc interactions. Along with discussing conjugation methodologies and radionuclide selections for created antibody radionuclide conjugates, we will also discuss future perspectives for the development and innovation of antibody radionuclide conjugates.

## 2 Construction of Antibody Radionuclide Conjugates

**Figure 1:**
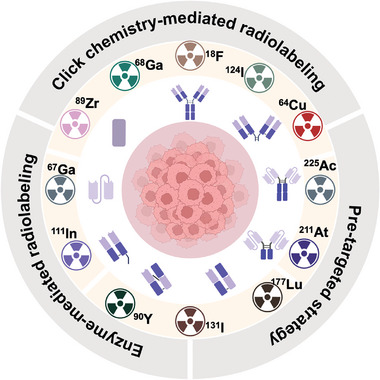
Overview of the antibody fragments, radionuclides, and conjugating methods discussed in this review.

The design and synthesis of ARCs have followed a consistent strategy since their inception. ARCs consist of three essential elements: an antibody that recognizes a tumor‐specific antigen, a radionuclide that delivers therapeutic or diagnostic radiation, and a connecting linker. However, the choice and optimization of each element can vary widely in ways that affect the pharmacokinetics and efficacy of ARCs (**Figure** **1**).^[^ ^27^, ^28^ ^]^

### 2.1 Engineered Antibodies

The term “antibody engineering” describes the process of changing the immunoglobulin scaffold using molecular biology methods to enhance the functionality of antibodies or radioimmunoconjugates. The humanization of antibodies is the most well‐known use of antibody engineering, and other excellent reviews have thoroughly examined the different techniques and uses of antibody engineering.^[^ ^29^ ^]^ Antibody engineering provides a means of optimizing radioimmunoconjugate performance for cancer radiotheranostics without the additional complexities of pretargeting or the drawbacks of antibody mimetics. Reducing the circulation half‐life to optimize the pharmacokinetics of antibodies, encouraging site‐specific bioconjugation, altering Fc interactions, or developing constructs with multiple target binding capabilities are the primary objectives of antibody engineering.

#### 2.1.1 Reducing the Circulation Half‐Life of Antibodies

The serum half‐life of a radioimmunoconjugate is determined by its interaction with the neonatal Fc receptor (FcRn), expressed in various cells and tissues throughout the body with a critical role in managing the immune system; FcRn binds to the IgG's Fc region of the radioimmunoconjugate, preventing its degradation by lysosomes. The FcRn also recycles IgG molecules back to circulation, thus extending the serum half‐life of the entire radioimmunoconjugate, allowing for high tumor uptake and prolonged radiation exposure. However, long‐lived radioimmunoconjugates also tend to circulate in the blood and deposit in healthy tissues, including the liver, spleen, and bone marrow,^[^ ^30^ ^]^ lowering the tumor‐to‐background (T/B) ratio.

**Figure 2:**
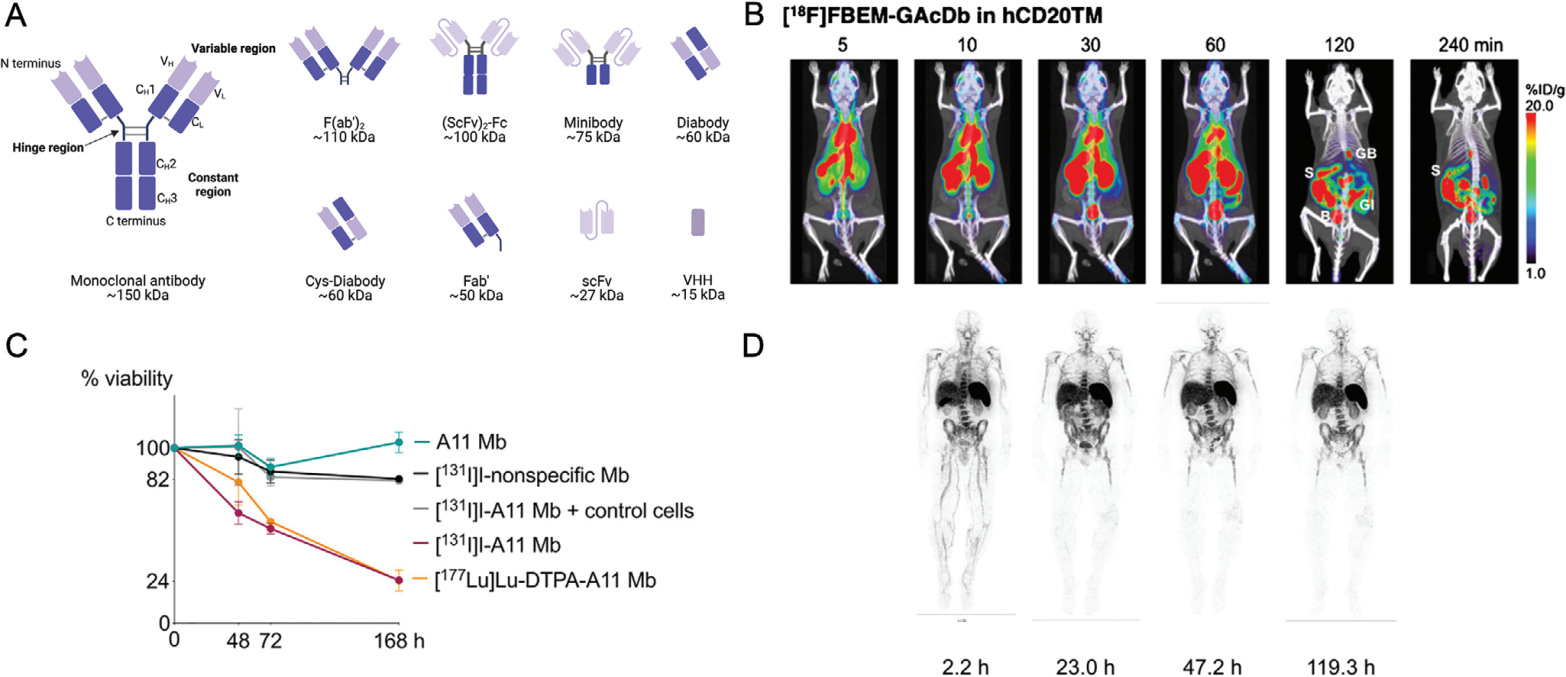
A) Commonly used antibody fragments. B) [^18^F]FBEM‐GAcDb immunoPET in human CD20 transgenic mice, 4.3 MBq/12 µg. The signal in the kidneys and the bladder increased over time indicating clearance/excretion of the tracer primarily through the kidneys and into the urine. Increased gallbladder (GB) and GI tract activities suggest secondary excretion of radio metabolites through the hepatobiliary system. Reproduced with permission.^[^ ^34^ ^]^ Copyright 2018, Springer Nature. C) [^131^I]I‐A11 Mb and [^177^Lu]Lu‐DTPA‐A11 Mb inhibit PSCA‐positive cell growth in vitro. Reproduced with permission.^[^ ^36^ ^]^ Copyright 2020, Springer Nature. D) Whole‐body images of 1 patient at various times after injection of ^89^Zr‐IAB22M2C (1.5‐mg minibody dose). All images show the most intense activity within the spleen, followed by marrow, liver, and kidneys. Reproduced with permission.^[^ ^40^ ^]^ Copyright 2020, SNMMI.

To increase the T/B ratio, antibody fragment constructs (**Figure** **2**A) have been utilized to increase tumor penetration due to their increased diffusivity and smaller size, which also facilitates the clearance of unbound radiotracers from the systemic circulation. Fragments also have different binding properties compared with whole antibodies, such as lower valency or affinity, that can reduce the binding site barrier effect, where high‐valency antibodies bind so effectively to antigens on the surface of tumors that they fail to penetrate deeply into the tissue, thus enhancing tumor saturation.^[^ ^31^, ^32^ ^]^ Moreover, lacking the Fc region, antibody fragments are potentially less immunogenic.

Some antibody fragments, including single‐domain antibodies (sdAb, ≈15 kDa; also known as VHH and nanobodies), single‐chain variable fragments (scFv, ≈27 kDa), F(ab) fragments (≈50 kDa), diabodies (≈60 kDa), minibodies (≈75 kDa), (scFv)2‐Fc fragments (≈100 kDa), and F(ab′)2 fragments (≈110 kDa) (Figure 2A).^[^ ^33^ ^]^ The choice of radionuclide affects the balance between therapeutic efficacy and safety, considering the pharmacokinetics of the antibody fragments and the half‐life of the radionuclides; the faster pharmacokinetics of fragments allows the use of shorter‐lived radionuclides (e.g., ^64^Cu, ^18^F, ^68^Ga, and ^211^At) instead of the longer‐lived ones (e.g., ^89^Zr, ^131^I, ^225^Ac, and ^177^Lu) typically used for radioimmunoconjugates. The goal is to optimize both the treatment impact and safety, as well as the logistical handling, including storage, transport, and disposal, which is easier with shorter half‐life radioisotopes. Because shorter‐lived radionuclides have lower radiation doses and shorter half‐lives, making them easier to handle and safer, this replacement improves the dosimetry and logistics of antibody fragments. Fragments also enhance image clarity and contrast while lowering background radiation. However, the quicker pharmacokinetics of antibody fragments come at the expense of decreased stability and affinity as compared to full‐length IgG. Radioimmunoconjugates based on antibody fragments usually have lower tumor uptake than those based on full‐length IgG, and some fragments show high renal uptake and retention during clearance, such as ScFv, which raises concerns when these antibody fragments are labeled with radioisotopes.^[^ ^22^ ^]^

Numerous preclinical studies have demonstrated the potential of antibody fragments for cancer radiotheranostics. Particularly for same‐day immunoPET, which involves imaging tumors that express certain antigens a few hours after injection using radiolabeled antibody fragments, such as diabodies or minibodies. Zettlitz et al. reported a cys‐diabody targeting CD20 in 2019 for same‐day immunoPET of B‐cell lymphoma. Using transgenic mice expressing human CD20 on mature B cells, they demonstrated the ability of an ^18^F‐labeled diabody, [^18^F]FB‐GAcDb, to identify both cancerous and normal B cells in the liver (Figure 2B).^[^ ^34^ ^]^ In 2023, Jonatan et al. used a ^68^Ga‐labeled anti‐CD70 VHH with a C‐direct‐tag with a free thiol to facilitate site‐specific conjugation to a NOTA bifunctional chelator, CD70 in human cancer xenografts.^[^ ^35^ ^]^ The tracer demonstrated the ability to distinguish and measure CD70‐positive tumors, indicating that a comparable imaging strategy might be applied in the clinic to pinpoint and monitor patients who will most likely benefit from anti‐CD70 treatment over an extended period. Shifting to radioimmunotherapy, Tsai et al. assessed an anti‐prostate stem cell antigen (PSCA) minibody (A11 Mb) tagged with ^131^I and ^177^Lu in a human prostate cancer xenograft model for pharmacokinetics and therapeutic effectiveness. The ^177^Lu‐labeled minibody ([^177^Lu]Lu‐DTPA‐A11 Mb) provided a lower radiation dosage to the tumor than the ^131^I‐labeled minibody ([^131^I]I‐A11 Mb), and the minibody demonstrated quicker clearance from blood and normal tissues than full antibodies. A single dosage of [^131^I]I‐A11 Mb used in radioimmunotherapy demonstrated dose‐dependent tumor suppression with low toxicity and improved survival, suggesting that the minibody might be a good vehicle for beta‐emitting isotope‐based targeted radionuclide therapy (Figure 2C).^[^ ^36^ ^]^ In another study, Feng et al. reported a radioimmunotherapy study using a HER2‐specific sdAb conjugated to a residualizing ^131^I‐labeled prosthetic group for HER2‐positive breast and ovarian cancer models. The radioimmunoconjugate [^131^I]SGMIB‐VHH‐1028 achieved a tumor‐to‐kidney therapeutic index of over 8.5 and almost completely inhibited tumor growth in BT474 xenograft‐bearing mice after doses of 18 and 30 MBq.^[^ ^37^ ^]^ The same group also demonstrated the efficacy of HER2‐targeted radioimmunotherapy using a conjugated sdAb to a residualizing prosthetic group that was identical and labeled with the alpha‐emitting radio halogen ^211^At.^[^ ^38^ ^]^

These preclinical findings are promising, but what is even more remarkable are the few clinical trials that have been conducted recently. In 2022, Morris et al. compared the performance of ^89^Zr‐df‐IAB2M, an ^89^Zr‐labeled anti‐PSMA minibody, and ^68^Ga‐PSMA‐11, a ^68^Ga‐labeled PSMA small molecule ligand, for PET imaging of localized prostate cancer, and correlated the results with multiparametric MRI. The PET tracers showed higher detection rates of prostate cancer than MRI, with ^89^Zr‐df‐IAB2M being superior to ^68^Ga‐PSMA‐11 in terms of sensitivity, specificity, and accuracy. The PET tracers also provided additional information about the presence and location of the therapeutic target, which could have implications for management change in men with localized prostate cancer.^[^ ^39^ ^]^ Additionally, the CD8‐targeting [^89^Zr]Zr‐IAB22M2C minibody‐based probe was recently clinically translated to image CD8‐positive T cells in a phase I trial. The results demonstrated that [^89^Zr]Zr‐IAB22M2C was safe, well‐tolerated, and could effectively target CD8‐rich organs such as the spleen and lymph nodes (Figure 2D).^[^ ^40^ ^]^ Moreover, Scott et al. reported on the use of a PEGylated ^124^I‐labeled diabody ([^124^I]I‐PEG‐AVP0458) for tumor‐associated antigen TAG‐72 PET imaging in patients with primary prostate cancer, metastatic prostate cancer, or ovarian cancer.^[^ ^41^ ^]^ As early as one day after injection, the radiotracer showed strong specificity and sensitivity for TAG‐72‐positive tumors, suggesting its potential as a diagnostic tool for cancer staging and monitoring.

Early‐phase clinical trials have proven the benefits of minibodies and other antibody fragments, which include lower immunogenicity and enhanced tumor targeting. However, obstacles, including intricate manufacturing procedures that raise production costs impede their wider clinical applicability.^[^ ^42^ ^]^ Furthermore, compared to full‐length antibodies, these fragments frequently show decreased stability and shelf life, which presents logistical difficulties. Clinical acceptance of innovative medicines is further delayed by the rigorous and time‐consuming regulatory routes.^[^ ^43^ ^]^ Integration into clinical practice is further delayed by the necessity for substantial clinical evidence to prove effectiveness and safety over current medicines. Despite these challenges, minibodies hold significant promise for targeted therapy. Continued research and development may resolve these problems and pave the way for broader clinical applications in the future.

#### 2.1.2 Site‐Specific Bioconjugation

Radioimmunoconjugates are designed to enable precision medicine, but their synthesis is surprisingly imprecise. Most radioimmunoconjugates are made by randomly attaching amine‐reactive prosthetic groups, such as chelators or radio‐halogenated moieties, to lysines in the mAbs. This method is easy, but it creates heterogeneous products and can affect the mAb's antigen binding.

Variability in conjugation methods can significantly influence the distribution, targeting accuracy, safety, and efficacy of radioconjugates. The pharmacokinetics, stability, tolerability, and effectiveness of early‐generation ARCs, for example, were shown to be suboptimal when they were manufactured as heterogeneous mixtures.^[^ ^44^ ^]^ Consequently, the focus was switched to creating homogenous constructs with precise drug loading and regulated attachment sites. When considering their pharmacological characteristics, homogeneous ARCs have proven to have better results than their heterogeneous counterparts.^[^ ^45^ ^]^ Additionally, by altering an amino acid's microenvironment, site‐specific bioconjugation can be accomplished, making it possible to activate a particular amino acid residue when other reactive species are present. This precision is crucial for maintaining the functionality of the mAb and ensuring the effective delivery of the therapeutic agent to target cells.^[^ ^45^ ^]^

There are instances of monoclonal antibodies where the effect on antigen binding has affected the effectiveness of treatment. For example, Entyvio (vedolizumab) is used to treat inflammatory bowel disease (IBD), while Avastin (bevacizumab) is used to treat colon cancer. Since these medications are made to attach to particular antigens, modifications to this capacity may have an impact on the therapeutic efficacy of the medications.^[^ ^46^ ^]^

The integrity of the mAb's antigen‐binding site must be preserved for site‐specific bioconjugation to result in the intended therapeutic effect. The homogeneity and effectiveness of these medicinal compounds are constantly being enhanced by developments in bioconjugation procedures.

To overcome these problems, various site‐specific and site‐selective bioconjugation methods have been developed, and the results demonstrate that they generate radioimmunoconjugates that function better in vivo than those generated at random.^[^ ^47^ ^]^ Precision is even more important for fragment‐based radioimmunoconjugates because their small size makes it more likely that the cargoes will interfere with their antigen‐binding domains. The most common site‐specific bioconjugation method uses thiol‐reactive probes, such as maleimide, to link to cysteines created by reducing the antibody's interchain disulfide bonds. This method is better than the traditional one, but it still has some drawbacks: the maleimide–thiol bond can break under physiologic conditions, and the full‐length IgG can have 4–8 free cysteines (depending on the reduction conditions), which can cause some heterogeneity. Therefore, the field has increasingly used antibody engineering to achieve more stable and consistent site‐specific bioconjugation.

**Figure 3:**
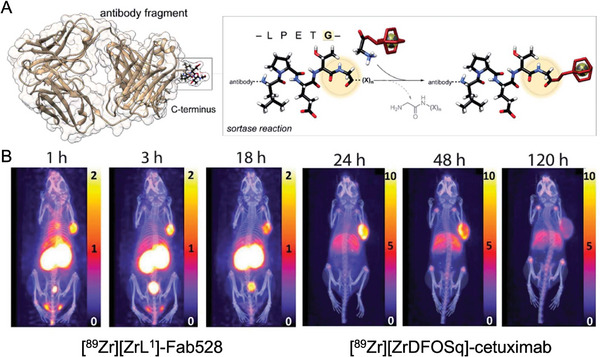
A) Schematic representation of the enzyme‐mediated bioconjugation of a chelator to a Fab using SrtA at the C‐terminal recognition sequence (LPETG). The threonine–glycine link is broken by the enzyme upon recognition of the short –LPETG– amino acid motif, and a cysteine residue found in the active site is converted to a thioacyl intermediate. The enzyme subsequently creates a new amide bond by accepting an incoming nucleophilic N‐terminal glycine. It is possible to modify the C‐terminus of the substrate protein by sourcing the incoming glycine from either the cleaved peptide or another peptide in solution that has the necessary N‐terminal glycine. B) PET/CT MIPs (scale given in SUV) following administration of [^89^Zr][ZrL1]‐Fab528 and [^89^Zr][ZrDFOSq]‐cetuximab. Reproduced with permission.^[^ ^48^ ^]^ Copyright 2021, RSC.

Peptide recognition sites can be added to immunoglobulins for chemoenzymatic labeling by antibody engineering. Rudd et al., for instance, employed the transpeptidase sortase A to bind glycine‐linked chelators to an epidermal growth factor receptor (EGFR)–specific Fab with a C‐terminal LPETG motif in a mouse model of EGFR‐positive epidermoid cancer. This resulted in ^64^Cu‐ and ^89^Zr‐labeled radioimmunoconjugates with very promising in vivo efficacy, demonstrating rapid tumor uptake, high tumor‐to‐background ratios at 1 h post‐injection of 7.54 ± 2.16 and 5.50 ± 0.30 respectively, and tumor retention time of up to 18 h, showing superior in vivo efficacy compared to the radiolabeled full antibodies that require several days between injection of the tracer and imaging (**Figure** **3**).^[^ ^48^ ^]^ Similarly, Bridoux et al. joined an hPD‐L1‐binding sdAb with a C‐terminal LPETG element to a NOTA variant with a GGGYK tag. They demonstrated that the in vivo performance of the site‐specifically labeled radioimmunoconjugate, [^68^Ga]Ga‐NOTA‐(hPD‐L1), was superior to that of its randomly labeled counterpart.^[^ ^49^ ^]^

Bioconjugation of antibodies with natural or unnatural amino acids at specific sites is also a promising strategy, especially for fragment‐based probes. For instance, Chigoho et al. made a radiotracer, [^68^Ga]Ga‐NOTA‐mal‐hPD‐L1, by attaching a maleimide‐linked NOTA variant to a C‐terminal cysteine of an hPD‐L1–binding sdAb, and showed its promising in vivo performance.^[^ ^50^ ^]^ A far more complex method introduces artificial amino acids with orthogonal reactive groups through genetic code expansion, providing unparalleled site selectivity. Using this technique, Ahn et al. added four p‐azido‐methyl‐phenylalanine residues to the mAb trastuzumab's Fc region.^[^ ^51^ ^]^ Next, they attached the antibody to DFO and DO3A that had been changed by dibenzocyclooctyne. The site‐specifically modified immunoconjugates were then tagged with ^89^Zr and ^111^In, respectively.

Interest in engineering‐driven bioconjugation has increased due to the chemoenzymatic bioconjugation. Vivier et al. truncated the heavy chain glycans of the HER2‐targeting antibody pertuzumab using the enzymes β‐galactosidase and endoglycosidase and then linked azide‐modified galactose residues to the residual sugars. They next added the chelator desferrioxamine (DFO) to the azide‐bearing glycans using the strain‐promoted azide‐alkyne click reaction, resulting in site‐specifically labeled immunoconjugates containing ^89^Zr. They showed that in a humanized mouse model of HER2‐positive breast cancer, the site‐specific bioconjugation not only increased the homogeneity and repeatability of the radioimmunoconjugates but also decreased their binding to human and murine FcγRI and improved their tumor uptake and contrast.^[^ ^52^ ^]^

#### 2.1.3 Modulating Fc Interactions

The pharmacokinetics and pharmacodynamics of mAbs are influenced by their Fc regions and how they bind to Fc receptors. While FcRn regulates the serum half‐life of antibodies, different types of Fcγ receptors—such as FcγRI, FcγRII, FcγRIIIA, and FcγRIIIB—modulate the immune response by forming immune complexes with antigens. Therefore, Fc engineering is a promising strategy to optimize the performance of radioimmunoconjugates for imaging and therapy.

**Figure 4:**
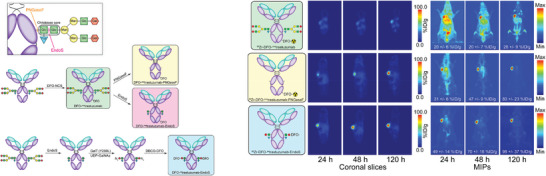
The preparation of the trastuzumab immunoconjugates. PET images of ^89^ZrDFO‐nsstrastuzumab, ^89^Zr‐DFO‐sstrastuzumab‐EndoS, and ^89^ZrDFO‐nsstrastuzumab‐PNGaseF in NSG mice bearing subcutaneous BT474 xenografts at 24, 48, and 120 h post‐injection. Reproduced with permission.^[^ ^55^ ^]^ Copyright 2019, SNMMI.

**Figure 5:**
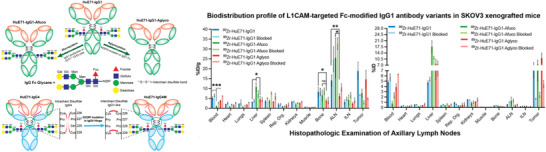
Scheme describing the generation of Fc variants of humanized IgG1 and hinge mutated IgG4 L1CAM‐targeted antibodies. Delineation of differential in vivo profiles of Fc‐modified L1CAM‐targeted IgG1 variants in SKOV3‐xenografted mice through *ex vivo* biodistribution analysis. Reproduced with permission.^[^ ^56^ ^]^ Copyright 2022, SNMMI.

Several studies have sought to modulate the behavior of mAb‐based radioimmunoconjugates by either increasing or decreasing Fc receptor binding. By interacting with Fc gamma receptors (FcγR), which are expressed in several organs, particularly liver Kupffer cells, the Fc region of mAb can cause off‐target binding. Certain antibodies are designed with mutations in the Fc region that decrease Fc‐receptor binding in order to overcome this. It was demonstrated by Mangeat et al. that in tumor‐bearing mice, the insertion of the LALAPG triple mutation in [^89^Zr]Zr‐DFO‐antibodies against several targets reduced FcγR binding and liver accumulation.^[^ ^53^ ^]^ They noticed that compared to wild‐type antibodies, the Fc‐engineered antibodies showed greater tumor‐to‐liver ratios and decreased liver absorption. These modifications were linked to improved Fc‐engineered antibody tumor targeting and imaging. Similar outcomes were noted by Burvenich et al. when using anti‐Lewis‐Y mAbs tagged with ^111^In and ^177^Lu, which had two mutations (I253A and H310A) that decreased FcRn binding.^[^ ^54^ ^]^ The heavy chain glycans of mAb modulate their Fcγ receptor engagement, which inspired the development of glycoengineered radioimmunoconjugates. In contrast to completely glycosylated monoclonal antibodies, Vivier et al. showed that the enzymatic removal of glycans from [^89^Zr]Zr‐DFO‐trastuzumab decreased FcγRI binding and absorption in healthy tissues in tumor‐bearing NSG and huNSG mice (**Figure** **4**).^[^ ^55^ ^]^ They found that the deglycosylated immunoconjugates had impaired FcγRI binding in vitro and reduced off‐target uptake in vivo, especially in the liver and spleen. These reductions were accompanied by increased tumoral uptake and improved tumor‐to‐healthy organ contrast and PET image quality. Two glycoengineered variants of the L1CAM‐targeting radioimmunoconjugate [^89^Zr]Zr‐DFO‐HuE71 were used more recently by Sharma et al. (**Figure** **5**) to demonstrate the effectiveness of this strategy.^[^ ^56^ ^]^ The afucosylated version with greater FcγRIIIA binding displayed higher accumulation in the liver and lymphoid organs and decreased tumor uptake compared to the parent radioimmunoconjugate. The low tumor uptake was attributed to the liver being a major sink. In contrast, the aglycosylated version that did not bind the Fcγ receptor exhibited significantly less accumulation in the bone and lymph nodes than the original [^89^Zr]Zr‐DFO‐HuE71 formulation, Lastly, Bensch et al. recently reported results from a clinical trial using [^89^Zr]Zr‐lumretuzumab, a full‐length HER3‐targeting mAb glycoengineered to improve FcγRIIIA binding and antibody‐dependent cellular cytotoxicity (ADCC) for PET imaging to assess target distribution.^[^ ^57^ ^]^ ADCC is a critical immune response where immune cells, primarily NK cells, recognize and destroy antibody‐coated target cells. Unlike fragments, which often lack the Fc region and therefore do not recruit effector functions, full‐length antibodies can achieve immune‐mediated tumor cell killing through enhanced ADCC, a process depending on antibody interaction with the FcγRIIIA on NK cells.^[^ ^58^ ^]^ The observation from preclinical studies that enhanced FcγRIIIA binding could lead to decreased tumor uptake and increased liver and lymphoid organ uptake highlights a complex balance in designing radioimmunoconjugate agents based on full‐length antibodies.

To modify the distribution and immune response functions of radioimmunoconjugates, the distinct Fc interactions of the four IgG subclasses, IgG1, IgG2, IgG3, and IgG4, have also been utilized. Bicak et al. synthesized an^225^Ac‐labeled IgG3‐based radioimmunoconjugate of the hexokinase 2–targeting mAb hu11B6 for use in prostate cancer radioimmunotherapy.^[^ ^59^ ^]^ Their idea was that the increased complement activation and Fcγ receptor binding of the IgG3 scaffold would result in an enhanced anti‐tumor immune response by the active recruitment of effector cells, leading to enhanced radioimmunotherapy. Nevertheless, neither the R435H mutant variant restored FcRn binding nor the [^225^Ac]Ac‐hu11B6‐IgG3 had enhanced therapeutic effectiveness over [^225^Ac]Ac‐hu11B6‐IgG1. Sharma et al.’s study, which was previously mentioned, not only explored how modifications to the Fc region affect the biodistribution of antibodies targeting L1CAM in vivo, but they also investigated the choice of IgG subclass. ^89^Zr‐immunoPET was performed using two IgG4‐based radioimmunoconjugates that target L1CAM. Since this subclass generates fewer effector functions than other subclasses, potentially decreasing the risk of toxicity, it has drawn interest in the realm of immunotherapeutics.^[^ ^56^ ^]^ However, wild‐type IgG4‐based radioimmunoconjugate exhibited high levels of nonspecific renal absorption. An engineered variant with a modified hinge region S228P resulted in reduced renal uptake. The modification prevented in vivo Fab arm exchange, a characteristic of IgG4, allowing for a more stable structure similar to IgG1‐like interchain disulfides. Lastly, Man et al. went beyond the IgG isotype by examining the in vivo behavior of an IgE‐based anti‐CSPG4 antibody designed to trigger a more robust immune response using, monitored with immunoSPECT.^[^ ^60^ ^]^ In the absence of FcRn‐mediated recycling, the [^111^In]In‐IgE was eliminated from the blood of tumor‐bearing mice significantly more quickly than its [^111^In]In‐IgG counterpart. The faster blood clearance of [^111^In]In‐IgE led to tumor‐to‐blood activity concentration ratios that were equal to those of homologous IgG, although it accumulated considerably in the liver.

Single‐chain fusion proteins composed of Fc domains and antigen‐binding fragments have also been used as radioimmunoconjugates. These fusion proteins share several characteristics with full‐length mAbs, such as large size, multivalency, and FcRn interaction. The single‐chain structure, which does not require simultaneous expression of heavy and light chains, can be produced more easily. Rochefort et al. developed an antibody fragment, ^124^I‐labeled (scFv)2‐Fc, that targets CA19‐9 and possesses an H310A mutation that prevents FcRn binding for microPET imaging.^[^ ^61^ ^]^ They found that the fragment had a similar affinity as the intact antibody and that the mutated fragment had an increased blood clearance rate. Delage et al. described using a ^177^Lu‐labeled anti‐TEM‐1 scFv‐Fc fusion antibody (scFv)2‐Fc, 1C1m‐Fc for theranostics of a sarcoma model. 1C1m‐Fc exhibited modest uptake and tumor‐to‐background ratios in TEM‐1–positive xenografts, although they made no mention of Fc engagement or mutations.^[^ ^62^ ^]^

The process by which therapeutic antibodies deliver cytotoxic payloads into cancer cells is known as endocytosis, and it is essential for the therapeutic potential of both conjugated (e.g., drug‐conjugated) and naked (unconjugated) antibodies.^[^ ^63^ ^]^ The Fc region interacts with Fc receptors on leukocytes to elicit various effector functions, one of which is ADCC, a crucial cytolytic mechanism of natural killer (NK) cells. Research is ongoing to enhance the Fc region's interaction with Fc receptors, such as CD16A on NK cells. This is because the Fc region's affinity for Fc receptors can greatly influence the efficacy of monoclonal antibody therapies, including those involving RIT.

#### 2.1.4 Binding Multiple Targets

**Figure 6:**
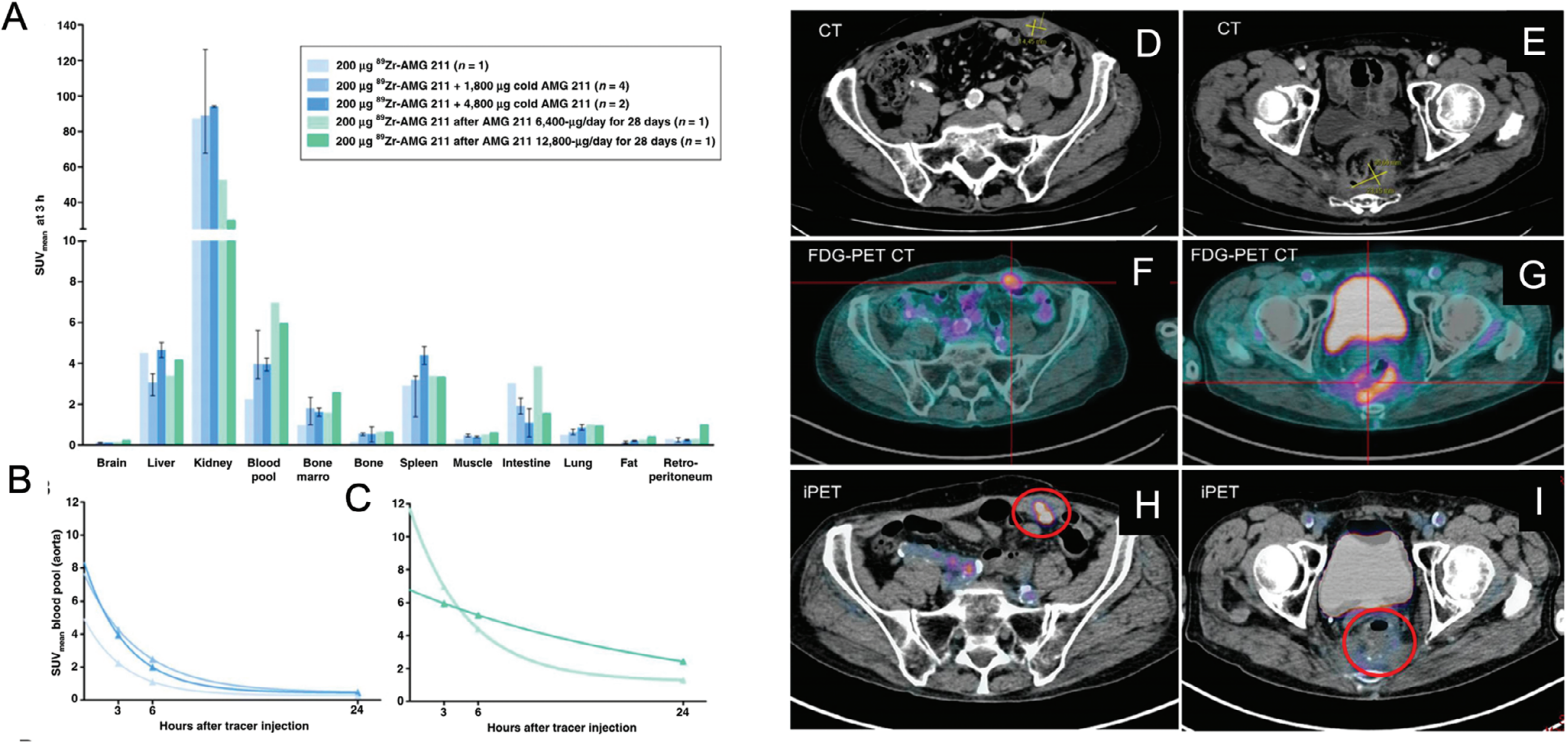
Biodistribution of ^89^Zr‐AMG 211. A) ^89^Zr‐AMG 211 healthy tissue biodistribution at 3 h post tracer administration for the different dosing cohorts used for imaging before (blue) and during (green) AMG 211 treatment. Data are shown as median SUVmean, error bars. B,C) Nonlinear regression curve showing mean SUVmean in the blood pool measured in the thoracic aorta per PET scan time B) before AMG 211 treatment and C) during AMG 211 treatment. Reproduced with permission.^[^ ^64^ ^]^ Copyright 2019, AACR. D) Imaging workup of patient 2 with a history of sigmoid cancer and synchronous liver metastases, treated with chemotherapy and intra‐arterial chemotherapy. E) Liver CT and MRI scans performed in the presence of a progressive increase in serum CEA show stable residual liver lesions with no evidence of tumor activity. F) FDG‐PET was negative. G–I) The immuno‐PET (iPET) revealed multiple liver tumor foci. Surgical specimens of the liver lesions confirmed the diagnosis of metastases from the sigmoid cancer. Reproduced with permission.^[^ ^69^ ^]^ Copyright 2020, Springer Nature.

Antibody engineering has enabled the development of radioimmunoconjugates that use bispecific antibodies (BsAbs) to recognize two different antigens simultaneously. Compared to traditional mAbs, BsAbs offer several benefits, including the ability to attract immune cells, overcome drug resistance, induce synergistic anticancer effects, and block protumor signaling pathways. Generally, a heavy‐chain/light‐chain pair from one mAb and another pair from a different mAb make up full‐length BsAbs (**Figure** **6**A). Various bi‐ and tri‐specific formats have been created using antibody fragments.

Like mAbs, BsAbs can be used as companion imaging agents for their cold equivalents. For example, immunoPET using ^89^Zr‐ labeled BsAb, which binds CD3 and carcinoembryonic antigen (CEA) to activate T cells in patients with gastrointestinal cancer, was studied in a clinical trial (Figure 6A–C).^[^ ^64^ ^]^ The CD3/CEA‐specific BsAb accumulated in tumor lesions as well as lymphoid organs, offering insights into the diversity of antigen expression. This data could be useful for designing patient‐tailored treatments and dosing regimens. Crawford et al. assessed the in vivo behavior of an alternative T‐cell‐engaging BsAb that targets CD3 and mucin 16 (MUC16) in a mouse model of ovarian cancer. They discovered that the organs with lymphoid function and tumor tissue had the highest activity concentrations.^[^ ^65^ ^]^ Blocking analysis, which demonstrated that blocking with a CD3‐specific mAb preferentially decreased uptake in the lymphoid tissues while blocking with a MUC16‐specific mAb did the same for the tumor, eloquently illustrated an explanation of this distribution.

BsAbs have also been used in the field to create radioimmunoconjugates that can pass through the blood‐brain barrier (BBB).^[^ ^66^ ^]^ The BBB normally prevents mAbs from passing through because of their large size and polarity, which limits the applications of radiolabeled antibodies in neuroimaging and therapy.^[^ ^67^ ^]^ However, it has been shown that tagging a transferrin‐binding Fab fragment to a BsAb facilitates the transferrin receptor‐mediated transcytosis of the bispecific construct across the BBB. Antibody fragments, with their high specificity and low background signal, provide enhanced imaging accuracy for brain targets compared to, e.g., small molecules, the lipophilicity of which enables them to cross the BBB but also contributes to their non‐specific binding. Syvänen et al. created a ^124^I‐labeled Tribody™ to identify amyloid‐β protofibrils in the brain, which are protein aggregates linked to Alzheimer's disease.^[^ ^68^ ^]^ They created five (A1‐A5) different fusion proteins that combine a transferrin receptor antibody with the amyloid‐β protofibril‐specific antibody mAb158. ^124^I‐A3 was shown on PET to only be retained in Aβ plaque pathology mice, with a region of interest to cerebellum ratio that increased from 1 at 2 h post‐injection to almost 3 at day 3 and correlated with *ex vivo* ELISA of soluble Aβ protofibrils.

Pretargeted imaging and therapy, decoupling the targeting of the tumor from the delivery of the radioactive payload to reduce the off‐target exposure and provide more favorable pharmacokinetics, is another way that BsAbs are used in nuclear medicine.^[^ ^22^ ^]^ The BsAb used in this method is developed to bind both an exogenous radiolabeled hapten and a tumor antigen. The unlabeled BsAb is injected first, giving the tumor time to absorb it from the circulation. Then, the high affinity and selectivity of the BsAb for the radiolabeled hapten, which is provided later, enable the in vivo ligation between the two components. This technique allows the use of short‐lived radionuclides that are generally incompatible with mAb‐based vectors while lowering radiation dose rates to healthy tissues. In two recent clinical investigations, pretargeted PET was evaluated in patients with breast and colon cancer using a ^68^Ga‐labeled peptidic hapten ([^68^Ga]Ga‐IMP288) and a BsAb (TF2) targeted to CEA. The results showed that this approach has faster clearance, lower radiation dose, and higher contrast than directly labeled antibodies (Figure 5D–I).^[^ ^69^, ^70^ ^]^

In general, radiolabeled antibodies for nuclear imaging and radioimmunotherapy have demonstrated excellent performance and adaptability through the application of antibody engineering for radiotheranostics. Nonetheless, certain challenges and limitations persist, necessitating focused attention on refining dosimetry and pharmacology, along with assessing safety and effectiveness in clinical studies.

### 2.2 Radionuclides

When selecting a radionuclide for conjugation to an antibody or a fragment, several parameters are taken into consideration. These include whether or not the protein will be internalized by the target cell, conjugation chemistry, and chelation procedure for radiometals. Once internalized radiometal‐based probes and their daughter isotopes, released from their chelators, are retained intracellularly because their polar and charged nature makes them unable to cross cell membranes, leading to accumulation in lysosomes.^[^ ^71^ ^]^ They can both enhance activity in normal tissue, particularly in excretory organs, and increase overall uptake in target tumor tissues. Conversely, free iodide or iodotyrosine released from radioiodinated internalizing carrier proteins are easily expelled from the cell and swiftly removed from the body. ^131^I is the most commonly used iodine isotope for SPECT and targeted radiotherapy of, e.g., NETs, prostate cancer, *etc*. While traditional treatment for thyroid cancer, especially differentiated thyroid cancer, relies heavily on radioiodine therapy without the need for carriers due to the thyroid's natural uptake of iodine, advancements in biotechnology have introduced various approaches for targeting thyroid cancer cells more specifically, particularly for types of thyroid cancer that are less responsive. For thyroid cancer, mAbs may be designed to target thyroid cancer cell‐specific markers, such as thyroglobulin and the thyroid‐stimulating hormone receptor. Conventional radioiodination methods like Iodogen lead to detachment of the isotope after internalization;^[^ ^72^ ^]^ overall, this leads to decreased background in imaging, but detached radioiodine can damage healthy thyroid cells similarly to free radioiodine therapy, and over time, internalization and degradation cause target (tumor) tissue to become less active.^[^ ^73^ ^]^ Thus, for internalizing radioiodine probes, using a different radioiodination method that ensures greater stability of the bond between iodine and the protein is more desirable. For probes that remain on the cell surface, radiopharmaceuticals designed for the radionuclide to detach or be released from their chelator after binding can be preferable because it allows potential irradiation of nearby tumor cells (cross‐fire effect), not just the cells to which the radiopharmaceutical is bound. This can be particularly useful in solid tumors, where not all cells might express the targeted antigen at levels sufficient for direct targeting. It is crucial to remember that internalization is not an all‐or‐nothing process; there is a broad range in the degree and rate of internalization of cell surface antigens. Some cell surface receptors and their bound antigens undergo a slow and continuous process of internalization, which is part of the natural membrane turnover. This steady‐state internalization allows for the maintenance of cellular functions and receptor homeostasis, ensuring that cells can respond to environmental signals over time without overaccumulation of specific receptors on the cell surface. In contrast, certain antigens, when bound to their receptors, can trigger a much faster internalization process, often observed in the context of receptor cross–linking, where the binding of an antigen (or an antibody) to a receptor leads to the aggregation of receptor‐antigen complexes which can activate signaling pathways that accelerate endocytosis.^[^ ^74^ ^]^

Common radionuclides that release positrons include ^18^F, ^68^Ga, ^64^Cu, ^89^Zr, and ^124^I; common radionuclides that release single photons are ^99m^Tc, ^123^I, ^131^I, and ^111^In (**Table** **1**). Shorter‐lived radionuclides (^18^F and ^68^Ga) match well with antibody fragments such as diabodies, single‐domain antibodies, and scFvs that have relatively short biological half‐lives, making them appropriate for imaging tumors a few hours after injection using radiolabeled antibody fragments. Longer‐lived radionuclides such as ^64^Cu, ^89^Zr, and ^124^I can mix well with the whole antibody or a larger antibody fragment such as the minibody for the greatest imaging contrast 24 to 48 h or more after injection.^[^ ^74^ ^]^

**Table 1:** Radionuclides used in antibody radionuclide conjugates.

Radionuclide	*T*_1/2_ [h]	Emission profiles	Other consideration	Reference
Single‐photon emitters		Principal γ energy [keV] [intensity %]		[112, 113]
^177^Lu	161	208 keV (11%)	β^−:^ (149 keV (80%)	
^67^Cu	62	184 keV (49%)	β^−:^ (1221 keV (57%)	
^123^I	13.1	159 keV (83%)	Auger: 3.2 keV (95%)	
^131^I	193	364 keV (81%)	β^−:^ 191 keV (90%)	
^111^In	67.2	245 keV (94%)	Auger: 2.8 keV (11%)	
		171 keV (91%)		
^99m^Tc	6.0	140 keV (98.6%)		
Positron emitters		Principal β+ energy (keV) (intensity %)		[114]
^68^Ga	1.1	836 keV (88%)		
^64^Cu	12.7	278 keV (18%)	β^−:^ (190 keV, 39%)	
^18^F	1.8	250 keV (97%)		
^89^Zr	78.5	396 keV (23%)		
_124_I	100.3	974 keV (11%)		

Positron yield, range, and other emissions falling within the scanner's energy range are additional factors to consider when choosing a radionuclide for imaging. For instance, the larger positron range of ^68^Ga and ^124^I compared to ^18^F results in reduced resolution and extra partial volume effects (PVE) that may have an impact on PET quantification.^[^ ^75^ ^]^ Some radionuclides, like ^64^Cu, have dual therapeutic and imaging properties because they decay and release high‐energy beta particles. To estimate the dosimetry of a therapeutic isotope, other positron emitters can be utilized as a surrogate, such as the pairing of the iodine isotopes ^131^I and ^124^I.^[^ ^76^ ^]^

RIT selectively delivers therapeutic radionuclides to the tumor site, but if the half‐life of the probe is too long, such as with intact antibodies, normal organs are unnecessarily exposed to radioactivity. Antibody fragments have the potential to lower the dosage to normal organs, particularly the radiosensitive bone marrow; nonetheless, renal elimination of fragment sizes necessitates monitoring the kidney dose.^[^ ^77^ ^]^ RIT radionuclides emit beta, alpha, or Auger electrons to cause cytotoxic DNA damage through a variety of processes, including the generation of reactive oxygen species, the breaking of single and double‐stranded DNA, and the inhibition of DNA repair (**Table** **2**).^[^ ^78^, ^79^ ^]^ Independent of the radionuclide, an immune response such as antibody‐dependent cellular cytotoxicity can also result in cell death, although the protein mass in RIT is frequently lower than a standard therapeutic antibody dosage. Target density, heterogeneity, and the kind and extent of the malignancy all impact the radionuclide selection because beta, alpha, and Auger emissions have different ranges and LETs.

**Table 2:** Radionuclides used in antibody radionuclide conjugates for RIT.

Radionuclide	*T*_1/2_	Maximum	Emission profiles	Other consideration	Reference
Beta emitters		Particle Range	Principal β^‐^ energy [keV] [intensity %]		[112, 113, 115]
^177^Lu	6.7 d	1.5 mm	149 keV (80%)	Imageable γ	
^131^I	8.0 d	2 mm	191 keV (90%)	Imageable γ Pairs with ^124^I PET	
^90^Y	2.7 d	11.0 mm	934 keV (>99%)	Bremsstrahlung: 0‐2.280 MeV	
^67^Cu	2.6 d	1.8 mm	1220 keV (57%)	Imageable γ pairs with ^64^Cu PET	
			468 keV (22%)		
			189 keV (20%)		
^47^Sc	3.3 d	0.2 mm	143 keV (68%)	Pairs with ^44^Sc PET	
			204 keV (32%)		
^166^Ho	1.1 d	8.7 mm	651 keV (50%)	Imageable γ	
			694 keV (49%)		
^161^Tb	6.9 d	1.8 mm	157 keV (65%)	Pairs with ^152^ Tb PET or ^155^ Tb SPECT	
			138 keV (26%)	Auger: 5.2 keV (88%)	
Alpha emitters			Principal α energy (keV) (intensity %)		[116]
^212^Bi	1.0 h	87 µm	6340 keV (35%)	1 α‐emitting daughter	
			6300 keV (26%)	1 β^‐^‐emitting daughter	
				Imageable γ	
^213^Bi	46 min	80 µm	8375 keV (98%)	2 α‐emitting daughters	
			5870 keV (2%)	2 β^‐^‐emitting daughters	
				Imageable γ	
^223^Ra	11.4 d	46 µm	5720 keV (52%)	3 α‐emitting daughters	
			5600 keV (25%)	2 β^‐^‐emitting daughters	
^221^At	7.2 h	55‐‐70 µm	5870 keV (42%)	4 α‐emitting daughters	
				3 β^‐^‐emitting daughters	
				Auger: 8.52 keV, 61.2 keV	
^225^Ac	10.0 d	85 µm	5830 keV (50%)	4 α‐emitting daughters	
			5790 keV (18%)	3 β^‐^‐emitting daughters	
				(1 daughter emits both α and β‐)	
				Imageable γ	
^149^Tb	4.1 h	25 µm	3970 keV (17%)	No α or β^‐^‐emitting daughters	
				Imageable β^+^	
Auger emitters			Principal Auger L, K energy (keV) (intensity %)		[115]
^67^Ga	3.3 d	10 nm	1.0 keV (168%)	Pairs with ^68^Ga	
			7.5 keV (61%)		
^125^I	60 d	10 nm	3.2 keV (157%)		
			23 keV (20%)		
^111^In	2.8 d	10 nm	2.8 keV (11%)	Imageable γ	
			20 keV (1.6%)		
^161 ^Tb	4.1 h	0.5‐‐30 µm	5.2 keV (88%)	Pairs with ^152^ Tb PET or ^155^ Tb SPECT	
			37 keV (1.5%)	Auger: 5.2 keV (88%)	

Due to the cross‐fire effect and the extensive reach of the radiation field impact spanning several millimeters, beta‐emitting radionuclides for RIT can influence tissue throughout and around the tumor attributed to their higher LET compared to gamma rays, typically ranging from 0.2 keV µm^−1^, and their relatively long path length in tissue, which can span from one to several millimeters. ^131^I is a common beta emitter used since 1941 for thyroid diseases and is readily available. In addition to RIT, ^131^I and ^177^Lu both release gamma photons that can be picked up by SPECT imaging. Antibodies coupled with ^177^Lu and ^90^Y catabolize the formation of charged radiometal chelate metabolites. To enhance tumor retention, these compounds are held onto in tumor cells. ^90^Y primarily emits beta radiation, not gamma rays, which conventional SPECT is designed to detect, which is one of its drawbacks. Conversely, ^90^Y generates Cerenkov radiation, a type of luminescent emission that occurs when charged particles like beta particles travel through a dielectric medium (such as biological tissue) at a speed greater than the phase velocity of light in that medium, and bremsstrahlung photons, which can be detectable by optical Cerenkov luminescence imaging and SPECT, respectively.^[^ ^80^ ^]^ Alpha emitters, such as the for RIT more frequently used ^225^Ac, ^211^At, ^213^Bi, and ^223^Ra can be helpful for treating smaller lesions and lesions resistant to beta radiation because of their high LET (80–100 keV µm^−1^) and much shorter range (a few cell diameters).^[^ ^81^ ^]^ In vitro and in vivo preclinical investigations have demonstrated the effectiveness of ^225^Ac‐labeled full‐length antibodies in killing tumor cells. ^225^Ac undergoes decay to generate four daughter particles that emit alpha radiation. Additionally, its decay chain includes the emission of SPECT‐visible gamma rays.^[^ ^82^ ^]^ However, the relatively low abundance and energy of gamma emissions in the decay chain of alpha particles, especially at the low therapeutic doses that patients receive, render SPECT ineffective for tracking their biodistribution.

Low‐energy Auger emitters, like ^125^I, are thought to have a high LET (4‐26 keV µm^−1^) and a very short path length in tissue (2–500 nm) (Table 2).^[^ ^83^ ^]^ Auger emitters, like alpha emitters, might be a better option than beta emitters for limiting damage to healthy tissues.^[^ ^84^ ^]^ Nonetheless, methods for intracellular delivery, ideally close to the cell nucleus, must be discovered.

Other radiometals, such as ^47^Sc, ^67^Cu, ^149^ Tb, ^161^ Tb, ^166^Ho, and ^212^ Pb, that may also be considered for antibody‐mediated imaging and treatment are listed in Table 1. Gamma rays and beta particles are released by a few of these radionuclides (^47^Sc, ^67^Cu, and ^166^Ho), which are useful for imaging and therapeutic purposes. ^149^ Tb produces alpha particles, positrons, and imageable gammas, making it useful for treatment as well as SPECT or PET imaging. These radionuclides have shown therapeutic potential in preclinical and clinical studies when labeled to full‐length antibodies and are, therefore likely to produce a similar effect with antibody fragment‐based RIT.^[^ ^85^ ^]^

### 2.3 Conjugation Strategies

One of the challenges in developing ARCs is the choice of an appropriate conjugation strategy that can attach radioactive isotopes to antibodies without compromising their stability, immunoreactivity, and pharmacokinetics.^[^ ^45^ ^]^ Conjugation to lysine residues is a common ADC method due to its convenience and stability; however, it lacks site‐selectivity, resulting in a mixture of products with variable properties and potential toxicity. For ARCs specifically, achieving a consistent and predictable biodistribution of the radiolabel is crucial for both therapeutic efficacy and minimizing radiation exposure to non‐target tissues. Therefore, methods that allow for site‐specific conjugation are being researched and developed, aiming to provide uniform radiotracers, ensuring that each antibody carries a predictable number of radioactive atoms, thus optimizing the balance between therapeutic effect and safety.^[^ ^86^ ^]^

#### 2.3.1 Enzyme‐Mediated Radiolabeling

Enzyme‐mediated radiolabeling, which employs enzymes to impart certain functional groups to antibodies that may react with radionuclide conjugates, is ideally suited to accomplish site‐specific labeling of antibody vectors. Enzymes are biocatalysts that can perform selective and mild reactions in biological systems. Site‐selective modification of antibodies via enzyme‐mediated radiolabeling can enhance the homogeneity, stability, and immunoreactivity of the radiolabeled conjugates.^[^ ^10^ ^]^

A recent study found that in immunoPET imaging investigations, site‐specifically modified ^89^Zr‐DFO‐trastuzumab showed enhanced immunoreactivity and stability, suggesting that it performed better than its counterpart created via a random conjugation approach. This resulted from using a chemoenzymatic approach. Site‐specific radiolabeling of VHHs with either ^18^F^[^ ^87^, ^88^ ^]^ or radiometals has been simplified by the use of sortase A (SrtA)^[^ ^89^, ^90^ ^]^ Additionally, a distinctive two‐step modular technique for conjugating immunoPET probes is available. In this method, SrtA is utilized to introduce strained cyclooctyne functional groups into the targeting vector of interest. With the development of SrtA variants that are Ca^2+^ independent and enhancements of SrtA's catalytic activity, SrtA will provide an adaptable platform for the creation of more complex immunoPET probes.^[^ ^75^, ^91^ ^]^

However, enzyme‐mediated radiolabeling also has some limitations, such as the need for pre‐functionalization of the radionuclide with the appropriate click handle, the potential interference of endogenous azides or alkynes in biological systems, and the availability and cost of the enzymes.^[^ ^92^ ^]^ To overcome these limitations, other enzymes that may change distinct amino acids or antibody patterns have also been used in various alternative techniques.

#### 2.3.2 Click chemistry‐Mediated Radiolabeling

**Figure 7:**
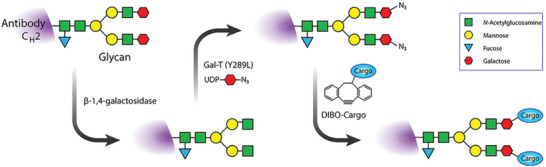
Schematic of a chemoenzymatic methodology for site‐specifically grafting cargoes (e.g., chelator) to the heavy‐chain glycans of an antibody of interest. Reproduced with permission.^[^ ^93^ ^]^ Copyright 2016, ACS.

Click chemistry‐mediated radiolabeling (**Figure** **7**) is a promising method that uses biorthogonal reactions to attach radioactive isotopes to antibodies that target specific tumor cells.^[^ ^93^ ^]^ Engineering techniques like inserting cysteine residues or unnatural amino acids enable bio‐orthogonal click chemistry. Click reactions encompass the inverse electron‐demand Diels‐Alder reaction (IEDDA) between tetrazine (Tz) and transcyclooctene (TCO), as well as the copper‐free strain‐promoted Huisgen cycloaddition.^[^ ^94^ ^]^ With first‐order rate constants as high as 10^5^ M^−1^ S^−1^, the IEDDA reaction 1,2,4,5‐tetrazine and strained alkene (such as TCO) is a well‐known biorthogonal reaction. It is usually considered the fastest click reaction.^[^ ^95^ ^]^ IEDDA has shown to be a very helpful ligation method for labeling radioisotopes with short half‐lives because of its exceptionally fast reaction rate in benign circumstances like room temperature, neutral pH, and aqueous medium. Additionally, tetrazines based on IEDDA‐labeled radioiodine can effectively solve the deiodination reaction caused by radiolabeled tracers based on the traditional radioiodination method by electrophilic substitution reaction. IEDDA‐based fast radiolabeling of antibodies was described by Valliant et al. To get the intended product in 69% radiochemical yield in this investigation, the TCO‐modified anti‐VEGFR2 was treated for 5 minutes with the ^125^I‐labeled tetrazine analog. It's interesting to note that the radiolabeled antibody produced by this method was 10 times more stable against in vivo deiodination than the identical antibody synthesized by direct radioiodination using iodogen.^[^ ^96^ ^]^

For diagnostic purposes, IEDDA ligation has also been used to generate a number of radioactive metal‐labeled tracers. Lewis et al. reported employing ^64^Cu or ^89^Zr to radiolabel norbornene carrying trastuzumab, together with tetrazine‐conjugated metal‐chelating agents such as DOTA and DFO.^[^ ^97^ ^]^ This process produced radiolabeled trastuzumab with high specific radioactivity (>2.9 mCi/mg) and high radiochemical yield (>80%). Additionally, PET imaging research indicated that radiolabeled antibodies exhibited specific absorption in HER2‐positive BT‐474 tumor cells and were rather durable in vivo. Therapeutic radioisotope‐labeled human antibodies 5B1 and huA33 were prepared in 2018 via IEDDA ligation.^[^ ^98^ ^]^ This work involved the synthesis of a tetrazine‐conjugated DOTA chelator tagged with ^225^Ac. Within five minutes, the TCO‐modified antibodies interacted with the radiolabeled tetrazine tracer to provide the intended products. Better radiochemical yields were obtained with this two‐step strategy compared with the traditional approaches used in clinical applications, The ^225^Ac‐labeled antibody also showed significant tumor uptake values and comparatively minimal non‐specific accumulation in normal organs, according to the biodistribution data.

#### 2.3.3 Pre‐targeting Strategy

**Figure 8:**
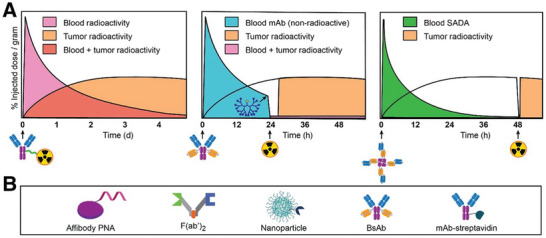
Comparison of conventional radioimmunotherapy and pretargeted radioimmunodetection/pretargeted radioimmunotherapy (PRID/PRIT) and compatible vector survey. A) Injection of radioimmunoconjugate (left) leads to low TIs, especially in hematopoietic and highly perfused tissues. With 3‐step BsAb pretargeting (middle), BsAb is administered, followed 1 d later by CA to quickly reduce circulating BsAb. During the final step, administered radiocarrier (e.g., radiohapten) is captured by intratumoral BsAb or rapidly cleared. A 2‐step approach (right) is feasible with SADA BsAb innovation. B) Representative bispecific antitumor/antiradiocarrier vectors. Reproduced with permission.^[^ ^99^ ^]^ Copyright 2022, SNMMI.

The goals of pretargeted radioimmunodiagnosis and radioimmunotherapy are to effectively combine therapeutic radioisotopes with anticancer antibodies for high‐contrast imaging and high‐therapeutic‐index (TI) tumor targeting, respectively (**Figure** **8**).^[^ ^99^ ^]^ Pretargeting strategies, in contrast to traditional radioimmunoconjugates, isolate the payload phase from the tumor‐targeting stage, increasing tumor uptake while minimizing exposure to normal tissue.^[^ ^23^ ^]^ A new BsAb platform for extremely effective 2‐step radio hapten pretargeting was described by Santich et al. as a combination of a tandem single‐chain BsAb and a self‐assembling‐and‐disassembling (SADA) domain.^[^ ^100^ ^]^ To increase the therapeutic index, they created a unique drug delivery platform that quickly eliminates tumor‐targeting proteins from the blood. The platform comprises a BsAb that binds to both ganglioside GD2 and DOTA, joined to a SADA domain. SADA–BsAbs were used for two‐step pretargeted radioimmunotherapy (PRIT) with various radioisotopes in mouse models of GD2‐positive neuroblastoma and GD2‐negative melanoma. The PRIT approach delivered high doses of radiation to tumors and eradicated them with negligible toxicity to the bone marrow, kidneys, or liver, demonstrating the potential of SADA–BsAbs as versatile and safe vectors for molecular targeting of cancer. The clinical trial of anti‐GD2 SADA‐PRIT and ^177^Lu‐DOTA‐hapten are planned to involve patients with malignant melanoma, sarcoma, and small‐cell lung cancer who express GD2 and are resistant or recurrent in their metastatic solid tumors (NCT05130255). The trial is currently recruiting participants and no results have been posted yet. Furthermore, a novel BsAb antitumor/anti‐chelate hapten pretargeting system (antigen targets: CD20, HER2, and CEA) based on an anti‐1,4,7,10‐tetrakis(carbamoylmethyl)−1,4,7,10‐tetraazacyclododecane (DOTAM) antibody with femtomolar affinity for lead‐DOTAM complexes was described by the teams at Hoffmann‐La Roche, Inc. and Orano Med LLC. In particular, they discovered that for lead‐DOTAM and bismuth‐DOTAM, the anti‐CEA/DOTAM BsAb PRIT‐0213 produced dissociation values of 0.84 pM and 5.7 pM, respectively. They reported dosimetry of 0.74 MBq of ^212^Pb‐DOTAM for three‐step pretargeting in nude mice carrying human cancer xenografts. They calculated an absorbed dose of 99.55 Gy to the BxPC3 tumor and TIs of 28, 14, and 91 for the liver, kidneys, and blood, respectively, based on relative biologic effectiveness of 5.^[^ ^101^ ^]^

#### 2.3.4 Combined Conjugation Strategies

Integrating different conjugation techniques offers distinct advantages for enhancing ARCs. Enzyme‐mediated conjugation alters antibodies at specific sites, avoiding random modifications and maintaining the antibody's affinity and functionality, but comes with limitations that can be overcome using copper‐free or catalyst‐free click reactions, including SPAAC or IEDDA.^[^ ^90^ ^]^ These methods offer advantages such as higher biocompatibility, faster kinetics, and lower background noise. The combination of enzyme‐mediated conjugation and click‐chemistry ensures that the conjugates are uniform and stable, improving the efficacy and safety of targeted therapies. The common glycosyltransferase‐mediated reaction involves the modification of the glycans on the antibody with specific functional groups, such as azide or alkyne, that can then react with the corresponding radionuclide conjugates via click chemistry.^[^ ^102^ ^]^ The glycosyltransferase‐mediated reaction can avoid the random modification of lysine or cysteine residues and produce more uniform and well‐defined ARCs. Zeglis et al., for instance, developed and verified a protocol for the enzyme‐ and click‐chemistry‐mediated site‐selective radiolabeling of antibodies on heavy chain glycans. Utilizing β−1,4‐galactosyltransferase (β4GalT1), a glycosyltransferase enzyme, they integrated azide‐modified N‐acetylgalactosamine monosaccharides into the antibody's glycans. Next, they clicked‐coupled desferrioxamine‐modified dibenzocyclooctynes to the sugars containing azide without the need for a catalyst. By using the positron‐emitting radiometal ^89^Zr to radiolabel the prostate‐specific membrane antigen‐targeting antibody J591, they were able to establish the antibody's high stability and immunoreactivity both in vitro and in vivo.^[^ ^102^ ^]^

When using pretargeting techniques, click chemistry facilitates a rapid and strong bond between the pretargeting and the radioactive components, enhancing the delivery of the radioisotope to the tumor site while minimizing exposure to healthy tissues. Bio‐orthogonal click reactions allow for quick radiolabeling (10–15 minutes), which is advantageous when using radionuclides with short half‐lives, such as ^18^F.^[^ ^91^ ^]^ Pretargeted ^18^F‐ and ^64^Cu‐PET imaging of in vivo models have been used to create high‐contrast imaging through the bio‐orthogonal click reaction between Tz and TCO.^[^ ^103^ ^]^ A pretargeted PET imaging technique based on the bio‐orthogonal Diels‐Alder click reaction between transcyclooctene and tetrazine was created by Zeglis et al. A ^64^Cu‐labeled tetrazine radioligand, and an anti‐CA19.9 antibody modified with transcyclooctene was utilized to image CA19.9‐expressing BxPC3 pancreatic cancer xenografts in mice. The pretargeting strategy demonstrated substantial tumor uptake (4.1 ± 0.3%ID/g) and tumor‐to‐background ratios of the ARCs at 4 h post‐injection while lowering the radiation dosage to normal tissues compared to directly labeled antibodies. Furthermore, the two arms of an anti‐EGFR and anti‐CD105 bispecific antibody were conjugated using click chemistry between Tz and TCO, and this antibody was then utilized for PET imaging. By connecting two antibody Fab fragments—an anti‐EGFR Fab and an anti‐CD105 Fab—via bioorthogonal “click” ligation of trans‐cyclooctene and tetrazine, Luo et al. created a bispecific immunoconjugate, known as Bs‐F(ab)_2_.^[^ ^104^ ^]^ They used a transcyclooctene‐modified anti‐CA19.9 antibody. Finally, pretargeted radioimmunotherapy investigations using TCO‐modified anti‐CA19.9 and ^177^Lu‐Tz radioligand have been conducted using click chemistry.^[^ ^105^ ^]^

Among different radiolabeling approaches, enzyme‐mediated radiolabeling excels in specificity but faces challenges in synthesis complexity,^[^ ^106^ ^]^ while click chemistry‐mediated radiolabeling is lauded for its efficiency and stable conjugate production,^[^ ^107^ ^]^ and pre‐targeting strategies are distinguished by their superior tumor targeting and reduced systemic toxicity.^[^ ^108^ ^]^ These approaches are complemented by the exciting fields of AI for predictive modeling and innovative BBB‐crossing techniques, which collectively signal a robust trajectory toward clinical application, as evidenced by recent literature.^[^ ^66^, ^108^ ^]^

## 3 Conclusion

Synthetic ARCs are a vital radiotheranostics tool with the potential to revolutionize cancer treatment. However, the majority of created ARCs have only been evaluated in small patient cohorts or during preclinical phases. To prove the theranostic utility of the clinically employed ARCs, and to translate some of the potentially designed ones into clinical use, more research is required. The development of new antibody therapies is transforming disease treatment, leading to the creation of more advanced antibody engineering techniques, including ARCs, which can aid physicians in improving their clinical judgment and realizing genuinely individualized medicine, ultimately benefitting the patient and society as a whole.

Non‐engineered antibodies like J591 continue to be widely used in clinical practice despite their known limitations. This prevalence is largely due to their long history of use, which has established a broad base of data supporting their safety and efficacy. Clinicians and patients alike tend to favor treatments with which they are familiar, and regulatory approvals for these antibodies further support their continued use.

On the other hand, engineered antibodies are increasingly recognized for their potential to overcome some of the limitations of traditional antibody‐based therapies. These modifications can improve pharmacokinetics, making them better suited for solid tumor imaging and treatment while maintaining the specificity and affinity characteristic of intact antibodies. However, engineered antibodies often face hurdles in clinical adoption due to ongoing clinical trials, limited availability, and higher production costs compared to their non‐engineered counterparts.

While further research is needed to fully establish the advantages of engineered antibodies, we believe they hold promise for addressing the shortcomings of conventional antibody therapies. As more data become available and these products reach regulatory approval, we anticipate an increase in their clinical application.

Additionally, certain challenges of all antibody‐based therapies regarding synthesis and delivery remain to be solved, and many approaches have been explored to this end. AI‐based antibody behavior prediction has the potential to be a key component in the behavior prediction of designed antibodies.^[^ ^109^ ^]^ Large‐scale information may be analyzed by machine learning algorithms to forecast the interactions between antibodies and various antigens. This capability can be very helpful in tailoring therapies for individual patients. Predicting the stability and effectiveness of antibodies under different physiological settings is another important aspect of AI's contribution to the success of antibody therapy.^[^ ^110^ ^]^

Furthermore, a major barrier to getting therapeutic medicines into the brain is the BBB.^[^ ^111^ ^]^ Cutting‐edge tactics to improve radioimmunotherapy agent distribution across the BBB are many and varied.^[^ ^67^ ^]^ First, nanocarriers can target brain tumors using radioactive isotopes that are encapsulated in them, thereby enabling larger concentrations of the therapeutic substance there with less systemic toxicity. Second, trojan horse approaches entail coupling antibodies to naturally bridging molecules like insulin or transferrin in order to expedite the delivery of therapeutic medicines to the brain. Last, by temporarily rupturing the BBB, osmotic agents or targeted ultrasound can facilitate the passage of antibodies.^[^ ^111^ ^]^

The development of mRNA‐based gene delivery platforms is at the forefront of translating engineered antibodies for use in clinical radioimmunotherapy. These platforms are necessary for the deployment of novel antibodies, such as transient, Fc‐free, multivalent, and multispecific variants. Major advancements include the development of chimeric antibodies that enhance immune responses against tumors and the clinical successes of bispecific antibodies for the management of diseases like rheumatoid arthritis and certain types of cancer. These developments are essential to advancing radioimmunotherapy from research to practical clinical settings and ushering in a new age in cancer therapy, together with AI's involvement in anticipating antibody behavior and creative methods for overcoming the blood‐brain barrier.

Undoubtedly, the preclinical evidence generated by numerous innovations, such as site‐specific bioconjugation techniques, sdAb‐based radioimmunotheranostics, and innovative approaches to pretargeted imaging and therapy, is cause for excitement. Moving these technologies from the lab to clinical settings is essential, and while it may seem easier said than done, generating clinical data as quickly as possible is what ultimately will advance their impact on patient care. Further promising preliminary research is likely to proliferate, especially as the cross‐pollination of ideas among immunotherapy, antibody‐drug conjugates, and radiotheranostics is further encouraged.

In both the therapeutic and diagnostic domains, antibody‐based radiopharmaceuticals have a promising future. Their ability to provide accurate imaging facilitates early disease diagnosis, which is essential for prompt treatment, and their capacity to deliver radioactive agents directly to cancer cells while sparing healthy tissue makes them promising therapeutic agents for targeted therapies with few side effects. Particularly exciting is the development of theranostics, which combines therapeutic and diagnostic properties in a single agent. This approach exemplifies individualized medicine, as it enables the simultaneous imaging and treatment of malignancies. The dual utility of antibody‐based radiopharmaceuticals is expected to increase as research advances, with theranostics setting the standard for innovation and providing a window into the future of integrated patient care. The advancement in this field is not about choosing between diagnostics and therapy but rather integrating both to enhance patient outcomes.

In summary, the future of modified antibodies for radioimmunotherapy will be characterized by a concentrated effort to improve site‐specific bioconjugation for increased safety and efficacy, adjust pharmacokinetics for improved tissue targeting, and modify Fc interactions to improve therapeutic indices. In addition, efforts are ongoing to identify novel molecular targets and develop bispecific constructs to improve targeting accuracy and reduce off‐target effects. Protein engineering developments will improve control over antibody properties, guaranteeing more efficient tumor delivery. Collectively, these research projects are paving the way for more individualized and effective radioimmunotherapy.

## Conflict of Interest

The authors declare no conflict of interest.
